# Elevated expression of periostin in diabetic cardiomyopathy and the effect of valsartan

**DOI:** 10.1186/s12872-015-0084-3

**Published:** 2015-08-18

**Authors:** Jun Guan, Wen-Qi Liu, Ming-Qing Xing, Yue Shi, Xue-Ying Tan, Chang-Qing Jiang, Hong-Yan Dai

**Affiliations:** Department of Cardiology, Qingdao Municipal Hospital, Qingdao, Shandong China; Qingdao University Medical College, Qingdao, Shandong China; Department of Clinical laboratory, Qingdao Municipal Hospital, Qingdao, Shandong China; Key Laboratory of cellular transplantation , Chinese Ministry of Public Health, Qingdao Municipal Hospital, Qingdao, Shandong China; Department of pathology department, Qingdao Municipal Hospital, Qingdao, Shandong China

## Abstract

**Background:**

Periostin, an extracellular matrix protein, plays a significant role in adverse cardiac remodeling. However, no report has documented the function of periostin in left ventricular remodeling of streptozototin (STZ)-induced diabetic rats. The aim of the present study was to observe the expression of periostin in Wistar rat’s myocardium of diabetic cardiomyopathy (DCM) and the effect of valsartan on it.

**Methods:**

Immunohistochemistry, real-time polymerase chain reaction, and Western blot analysis were used to determine the degree of expression and location of periostin, transforming growth factor (TGF)-β1, TGF-β1 type II receptor (TGF-β1 R II), and Type I and III collagens in the myocardium of STZ-induced diabetic rats.

**Results:**

Periostin, TGF-β1, TGF-β1 R II, and Type I and III collagens were significantly increased in the myocardium of diabetic rats compared with control group on both messenger ribonucleic acid and protein levels. In addition, diabetic rats treated with valsartan could have reduced expression of periostin and improved cardiac remodeling of DCM.

**Conclusions:**

Periostin may play a crucial role in cardiac remodeling and myocardial interstitial fibrosis process of DCM and it could be one of the important mechanisms for valsartan to improve the ventricular remodeling of DCM.

## Background

Diabetes mellitus remains a highly prevalent and a vigorous independent risk factor for cardiovascular disease. Recently, an accumulating number of evidence has demonstrated that the presence of abnormal myocardial structure and myocardial dysfunction in patients with diabetes in the absence of epicardial coronary artery disease (CAD), hypertension, and congestive heart failure after adjusting left ventricular hypertrophy [1–3]. This hypothesis was originally recognized in 1972 by Rubler S, who described data from patients with diabetes and heart failure without any evidence of CAD, valvular heart disease, hypertension, or congenital heart disease. This phenomenon has led to the increased recognition of a distinct disease process defined as “diabetic cardiomyopathy” (DCM). Multifactorial mechanisms were thought to be involved in the pathogenesis of DCM. Predominantly, myocardial fibrosis and myocyte hypertrophy are the significant mechanisms to explain the cardiac changes of DCM [4].

Periostin, a 90-KDa secretory protein within the extracellular matrix (ECM), has been derived from studies of bone development wherein it is expressed in both the periodontal ligament and the cortical bone periosteum of adult mice [5]. The association between periostin and myocardium was first reported in the context of mature valves and endocardial cushion development [6]. Recently, numerous studies have described a strong relationship between periostin overexpression and cardiac remodeling in human [7] and rat failing heart or myocardial heart [8–11]. Significantly, periostin is sensitive to transforming growth factor (TGF)-β activation and has emerged to play an important role in collagen fibrillogenesis [12–14]. All of these findings suggest that periostin is an important novel factor in the pathogenesis of cardiovascular disease and in the evolution of cardiac remodeling. Meanwhile in vitro study showed that high glucose increased periostin expression in adult rat cardiac fibroblasts [15]. So it is valuable to elucidate the expression level and possible function of periostin in DCM.

Valsartan, an Angiotensin II (Ang II) receptor blocker, has been reported to protect against diabetic cardiomyopathy [16]. Blockage of Ang II AT1 receptor is thought to be the main mechanism of valsartan’s protection on cardiac remodeling. But is there any other mechanism involved? In previous studies, it has been demonstrated the existence and activation of a cardiac renin-angiostensin system (RAS) in the hearts in diabetes [17]. Ang II could stimulate the periostin expression in both fibroblasts and cardiac myocytes in vitro, and this increase was inhibited partially, but significantly, by valsartan [11, 18]. Periostin can induce fibroblast proliferation and myofibroblast persistence in vitro [19]. In vivo, valsartan can also significantly attenuate the increased periostin expression, accompanied by improvement of cardiac dysfunction in acute myocardial infarction rat models [11]. But whether periostin takes part in the valsartan-induced protection of DCM is still unknown.

So it is worth clarifying the role of periostin in cardiac remodeling of DCM as no studies yet documented the relation between the periostin and DCM. In the present study, the changes of periostin expressions in cardiac remodeling of DCM and the effects of valsartan on the regulation of periostin expression were examined.

## Methods

### Induction of diabetes

Male healthy Wister-Albino rats (6-week-old; 180-220 g; n = 60) were purchased from the animal center of Lu-kang corporation. All experimental procedures were performed in accordance with the institutional animal care guidelines. Rats were housed in an air-conditioned room at a constant temperature of 20 ± 2 °C and were fed with a standard diet and water at liberty. Following one week of acclimatization, the rats were assigned randomly to a control group (n = 10) and a diabetic group (n = 50). All rats were weighed, then blood glucose levels were measured initially before the experiment and then weekly once throughout the experiment. Diabetes was induced by a single intraperitoneal injection of streptozotocin (STZ) (60 mg/kg body weight; Sigma, USA) dissolved in 0.1 mmol/L sodium citrate buffer (pH adjusted to 4.2), whereas rats in control group were injected with 0.1 mmol/L citrate buffer alone. All rats were given the same weight of normal (non-high fat and sugar) diet and the same volume of water. Three weeks after the injection of STZ, blood was collected from tail vein and samples were analyzed for blood glucose using a glucometer. Rats with random fasting blood glucose levels greater than 16.7 mmol/L were considered as the distribution criteria of type 1 diabetic rat. In addition, the eligible diabetic rats were randomly assigned to two groups: diabetes group (n = 21) and diabetes plus valsartan group (n = 20). Rats in diabetes plus valsartan group were given valsartan by gavage using a suitable intubation cannula at 30 mg/kg/day dose levels to soluble in 5 mL distilled water, whereas rats in the diabetes group were administered with the same amount of distilled water each day. No additional treatments were given to control rats.

After 16 weeks, all animals from each group were euthanized using pentobarbital and fresh hearts were harvested immediately. Then, weight of the left ventricle was noted in order to observe the degree of left ventricular hypertrophy. Furthermore, a transversal cross-section block was cut from the left ventricle, soaked in 4 % paraformaldehyde, and remained frozen in liquid nitrogen until mechanical testing.

### Hematoxylin eosin (HE) and Masson’s trichrome stain of myocardial tissue

After 48 h of treatment with 4 % paraformaldehyde, the tissues were dehydrated and the transparent tissues were embedded in paraffin. The tissues were cut into 6-μm slices, heated at 60 °C for 3 h, dewaxed, and stained with HE and Masson’s trichrome dyeing kits. Each slice was analyzed under a microscope at 400× magnification. The collagen volume fraction was determined as the ratio of interstitial collagen area to myocardial area.

### Immunohistochemistry

For immunohistochemical analysis, the transversal sections of the left ventricle were cut into 5-μm sections and the endogenous peroxidase activity was quenched by incubation with 3 % hydrogen peroxide for 15 min. The sections were then blocked using 5 % bovine serum albumin to prevent the nonspecific staining with the secondary antibodies. After overnight incubation at 4 °C with primary antibodies (anti-periostin, anti-TGF-β1, anti-TGF-β1 RII, and anti-collagen I and III antibodies were used at 1:100 dilutions), the sections were exposed to the secondary antibody conjugated with horseradish peroxidase. The reaction was visualized using a light microscopy with 3, 3’-diamno-benzidine tetrahydrochloride.

### Western blot analysis

Protein fractions were isolated in ice-cold radioimmunoprecipitation assay lysis buffer (Beyotime, China) and protein concentrations were determined using the enhanced bicinchoninic acid protein assay kit (Beyotime, China). Protein fractions were denatured in a loading buffer, and 100 μg of each sample was loaded into alternating lane of 10 % sodium dodecyl sulfate -polyacrylamide gel. Protein blots were transferred to polyvinylidene fluoride membranes (Milipore, USA). After blocking with 5 % non-fat milk, the blots were washed with a mixture of Tris-buffered saline and Tween 20 (TBST) and incubated overnight at 4 °C with an appropriate primary antibody (anti-β-actin, anti-periostin, anti-TGF-β1, anti-TGF-β1 II R, and anti-collagen I and III antibodies were used at 1:1000, 1:500, 1:400, 1:2000, 1:2000, and 1:5000 dilutions, respectively). Membranes were washed with TBST and then incubated at room temperature for 1 h with an appropriate secondary antibody conjugated to horseradish peroxidase and washed again. Finally, the protein blots were visualized using an enhanced chemiluminescence kit (Millipore, USA). Relative intensities of protein bands were measured using Gel-Pro analyzer. β-actin was used as a control.

### Real-time polymerase chain reaction (RT-PCR) analysis

The total RNA was extracted from the frozen myocardial tissues using Trizol reagent (Invitrogen, USA), according to the manufacturer’s instructions. The concentration of extracted total RNA was quantified using spectrophotometry. The reverse transcription of RNA to complementary deoxyribonucleic acid (cDNA) was performed using Sensiscript RT kit (TaKaRa Biotechnology, China). Total cDNA was amplified with LightCycler-FastStart DNA Master SYBR Green I (TaKaRa Biotechnology, China). Primers for rats periostin, TGF-β1, TGF-β1 R II, Type I collagen, Type III collagen, and β-actin were designed by TaKaRa Biotechnology Corporation: Periostin S: 5’-GGCTGAAGACTGCCTTGAATGAC-3’, A: 5’-CGTGGCAGCACCTTCAAAGA-3’; TGF-β1 S: 5’-AGGTAACGCCAGGAATTGTTGCTA-3’, A: 5’-CATTGCTGTCCCGTGCAGA-3’; TGF-β1 R II S: 5’-CTACAAGGCCAAGCTGAAGC-3’, A: 5’-AGCCAATGGAAGTAGACATCCG-3’; collagen I S: 5’-AGGGACCCTTAGGCCATTGTGTA-3’, A: 5’-GACATGTTCAGCTTTGTGGACCTC-3’; collagen III S: 5’-GACAGATCCCGAGTCGCAGA-3’, A: 5’-TTTGGCACAGCAGTCCAATGTA-3’; and β-actin S: 5’-AGACCTTCAACACCCCAG-3’, A: 5’-CACGATTTCCCTCTCAGC-3’. Amplification process included an initial denaturation step of 30s at 95 °C followed by 40 cycles of three-step procedure (denaturation at 95 °C for 5 s, annealing at 60 °C for 30s, and extension at 60 °C for 30s) and then a melting-curve procedure at 60 °C for 1 min. At the end of each cycle, fluorescence emitted by the SYBR Green I dye was measured. For each PCR product, a single narrow peak was obtained through melting curve analysis at a specific melting-curve temperature, indicating specific amplifications. The amount of periostin, TGF-β1, TGF-β1 R II, Type I and Type III collagens, and β-actin transcripts was calculated from their respective standard curves using the Light Cycler software. Samples were tested for three times, and the average values were used for quantification. Ultimately, the relative expression levels of each target gene were normalized to the messenger RNA (mRNA) of the internal standard gene β-actin.

### Statistical analysis

All data were expressed as mean ± standard error of the mean. Comparisons between two groups were performed using paired student’s t test. One-way analysis of variance was used to compare more than two groups. A value of P less than 0.05 was considered statistically significant.

## Results and discussion

### General characteristics of the experimental rats

**Fig. 1:**
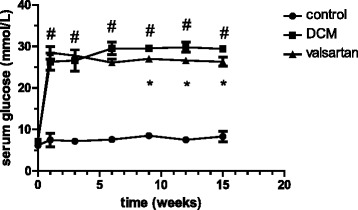
Serum glucose levels of experimental animals. The first week: control (7.513 ± 1.589) vs DCM (26.534 ± 0.684) #*p* = 0.0002. The third week: control (7.085 ± 0.527) vs DCM (25.940 ± 0.661) #*p* = 0.0001. The sixth week: control (7.703 ± 0.454) vs DCM (28.811 ± 0.448) #*p* = 0.0001. The ninth week: control (9.282 ± 0.167) vs DCM (29.441 ± 0.406) vs valsartan (27.303 ± 0.398) #*p* = 0.0001,*p = 0.0197. The twelfth week: control (7.948 ± 0.242) vs DCM (29.129 ± 0.591) vs valsartan (26.887 ± 0.832) #*p* = 0.0001, *p = 0.0332. The fifteenth week: control (8.949 ± 0.579) vs DCM (29.101 ± 0.599) vs valsartan (26.554 ± 0.542) #*p* = 0.0001, *p = 0.0347

At the end of the experiment, 21 STZ-induced diabetic rats, 20 diabetic rats with valsartan, and 10 control rats were survived, and the rest of 9 diabetic rats were died due to infection. Blood glucose levels were significantly increased in diabetic rats compared with control rats at the end of one week after STZ injection, the difference has statistically significant (*p* = 0.0002). Meanwhile, the glucose levels of diabetic rats were decreased by valsartan treatment at the end of nine weeks, the difference between diabetic group and diabetic plus valsartan have statistically significant (*p* = 0.0197) (see Fig. 1). The STZ-induced diabetic rats were at a diabetic state of polydipsia, polyphagia, and polyuria with lowered body weights.

### Degree of left ventricular hypertrophy

**Fig. 2:**
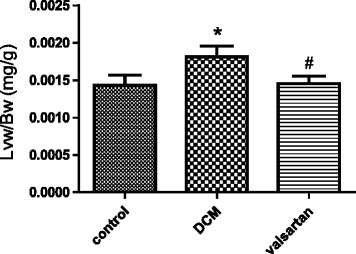
The degree of left ventricular hypertrophy of experimental animals. Control (1.43 ± 0.08) vs DCM (1.81 ± 0.8) vs valsartan (1.52 ± 0.55) #*p* = 0.0295, *p = 0.0427

Left ventricular coefficient, used to assess the extent of cardiac hypertrophy, which can calculated by comparing the left ventricular weight and body weight [20]. The left ventricular coefficient of diabetic rats was higher than control rats (*p* = 0.003), whereas it was lower in the valsartan-treated diabetic rats than the diabetic rats without valsartan (*p* = 0.006), those differences have statistically significant (see Fig. 2).

### HE and Masson’s stains of myocardium

**Fig. 3:**
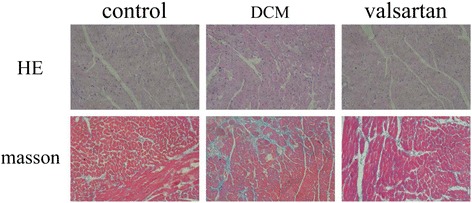
Hematoxylin and eosin stained (*purple* and *red*) rat’s myocardium. The cardiomyocytes size and quantity were increased in DCM rats compared with control rats, whereas it was obviously alleviated in the valsartan-treated diabetic rats than DCM rats (×400). Masson’s trichrome staining of rat’s myocardium revealed that the collagen deposition was obviously enhanced in the myocardium of diabetic rats than control rats; however, the myocardial fibrosis was markedly decreased in the valsartan-treated rats than DCM rats (×400)

Through light microscope, it was observed that the diabetic rats had increased sizes as well as disordered arrangement of cardiocytes compared with control rats, while the myocardial distribution of valsartan-treated group obviously improved than DCM group. The cardiac collagen deposition was dramatically increased in the diabetic rats than control rats. However, the amount of collagen in valsartan-treated rats was markedly alleviated than diabetic rats (see Fig. 3).

### Immunohistochemical analysis

**Fig. 4:**
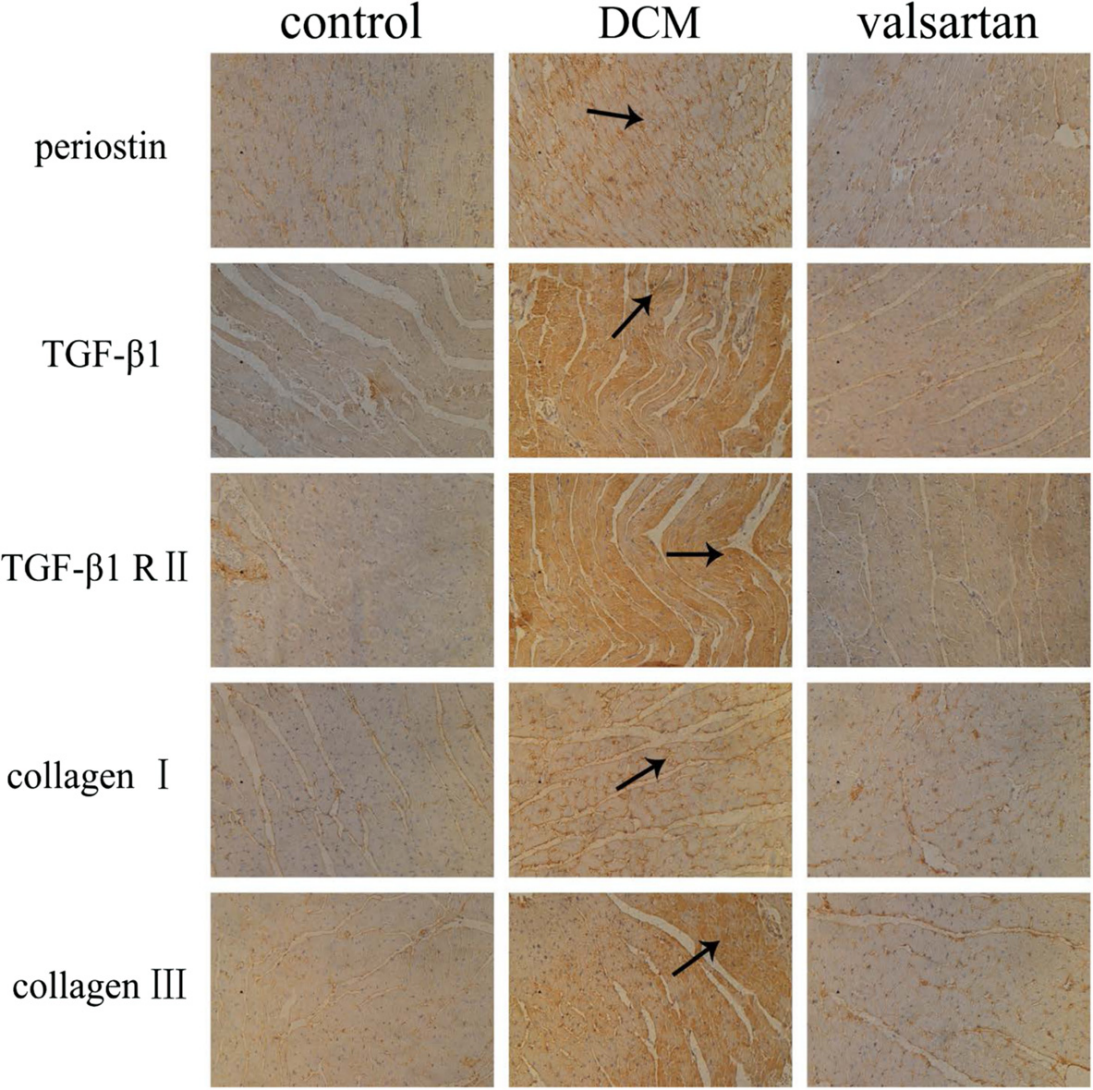
Immunohistochemical staining of periostin, TGF-β1, TGF-β1 R II, and Type I and III collagens in the control, DCM, and valsartan-treated group. The representative images of *brown* grain deposition showed the localization of the proteins. In contrast to the little or no protein staining in the control rat’s myocardium, the DCM rats displayed increased expression of above mentioned proteins in the heart, while the protein expressions were decreased in the valsartan-treated diabetic rats comparted to DCM group (×200)

Immunohistochemical analysis revealed that the enhanced staining of periostin occurred in fibroblast, endothelial cells, inflammatory cells, and myocytes in hearts of diabetic rats, whereas little periostin protein expression was found in the control rats. Similarly, weak collagen I and III protein expressions were found in the control rats, whereas the diabetic rats showed strong collagen deposition in hearts. Furthermore, strong TGF-β1 and TGF-β1 R II expressions were also obviously found in diabetic rats; however, there was little or no expression in the control rats. Moreover, in valsartan-treated diabetic group, the expression of periostin, TGF-β1, TGF-β1 R II, and Type I and III collagens were alleviated compared to DCM group (see Fig. 4).

### Western blot analysis

**Fig. 5:**
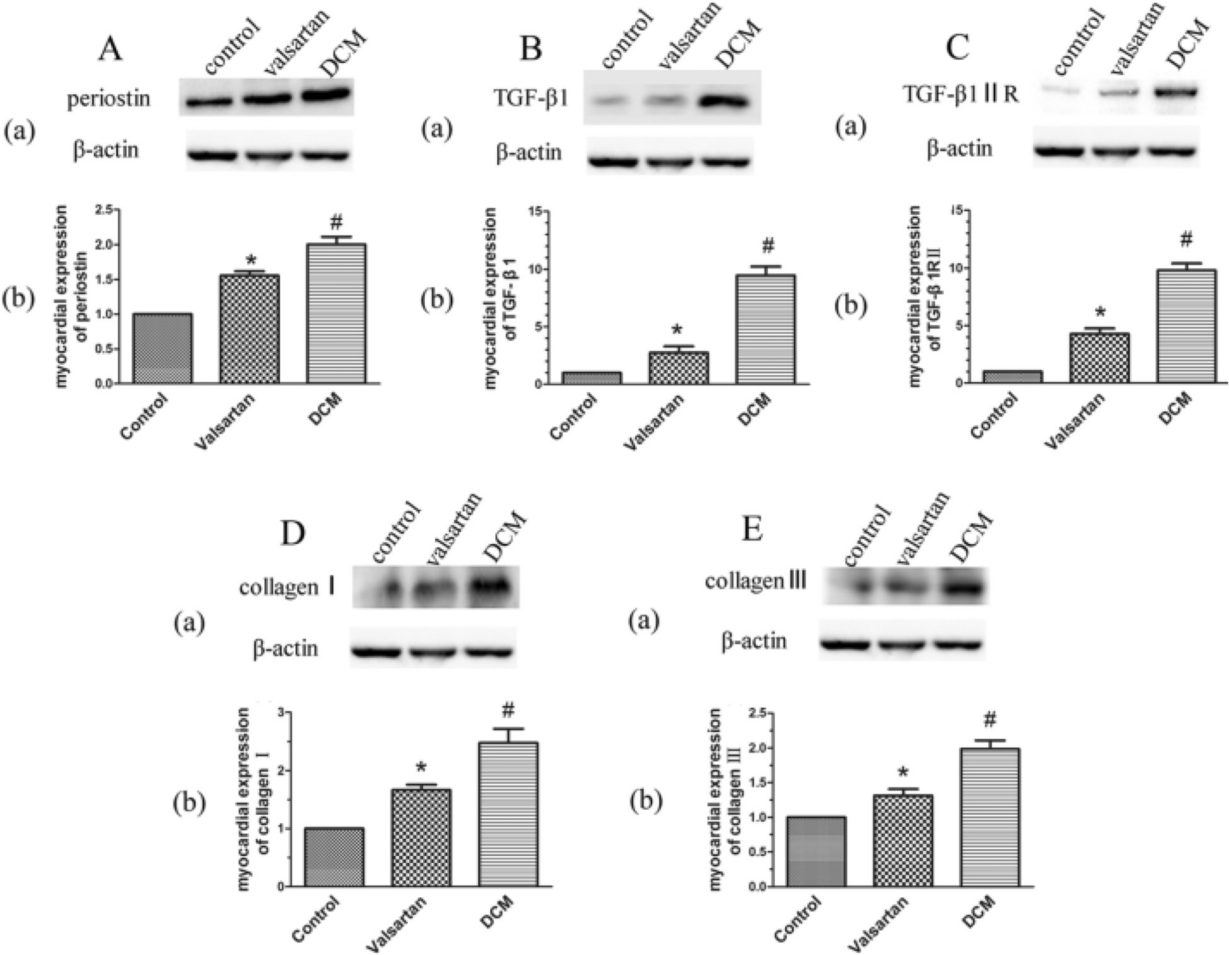
Western blot analysis of periostin (**A**), TGF-β1 (**B**), TGF-β1 R II (**C**), Type I (**D**) and III collagen (**E**) proteins expression in the hearts of control rats, valsartan-treated rats, and DCM rats. (a) The representative Western blot images demonstrated these protein expression levels in three groups. (b) Graph demonstrating quantification of these proteins in Western blot. DCM group showed obviously enhanced proteins expression of periostin, TGF-β1, TGF-β1 R II, Type I and III collagen in hearts compared to control rats. The expression of proteins was significantly alleviated in the valsartan-treated group than DCM group. Data were shown as mean ± SEM. #*P* < 0.05 vs. control; *P < 0.05 vs. DCM

The present study results revealed that the expression of periostin was extremely upregulated in DCM rats compared to control rats. And the overexpression of periostin was inhibited by the valsartan treatment. In addition, TGF-β1, TGF-β1 R II, and Type I and III collagens were regarded as a marker reflecting the activation of cardiac fibrosis [21, 22]. All these markers were expressed abundantly in the hearts of diabetic rats compared with control rats, and all these marker expressions were reduced after the valsartan treatment (see Fig. 5).

### Myocardial mRNA expression

**Fig. 6:**
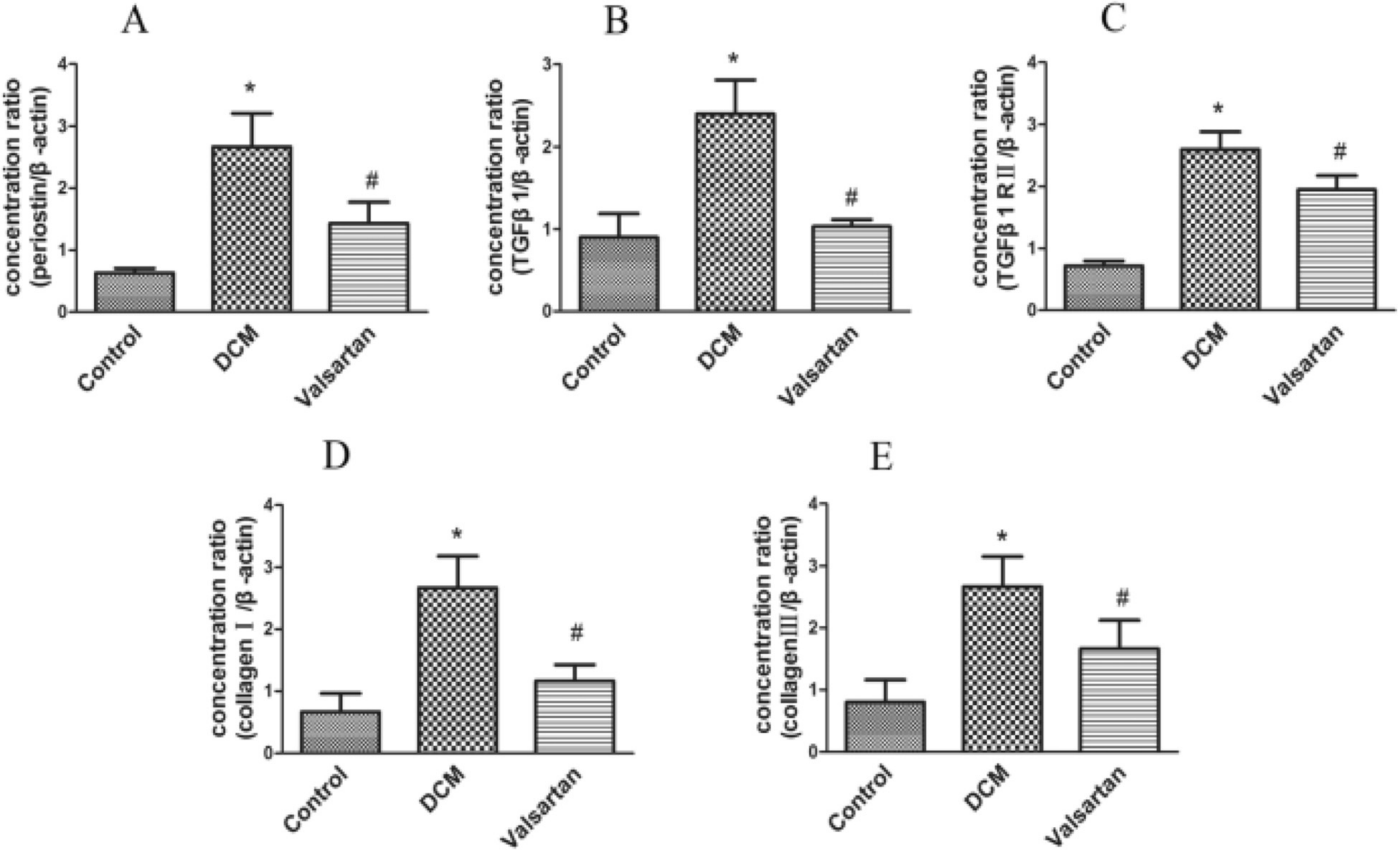
Real-time polymerase chain reaction (RT-PCR) analysis of periostin (**a**), TGF-β1 (**b**), TGF-β1 R II (**c**), Type I (**d**), and Type III collagens (**e**) mRNA expressions in the hearts of control rats, DCM rats, and valsartan-treated diabetic rats. DCM group showed significantly enhanced periostin, TGF-β1, TGF-β1 R II, Type I and III collagen mRNA expressions compared with control rats, while in valsartan-treated diabetic group, the mRNA expressions were decreased compared to DCM group. A total of six samples for each group were used, and each sample was run in triplicate for real-time PCR. Data were shown as mean ± SEM. *P < 0.05 vs. control; #*P* < 0.05 vs. DCM

The RT-PCR analysis revealed that a remarkable increase in periostin mRNA expression in the diabetic rats compared with control rats. In addition, the mRNA expressions in valsartan-treated rats were significantly decreased compared to diabetic rats after 8 weeks. Furthermore, TGF-β1, TGF-β1 R II, and Type I and III collagen mRNA expressions in DCM group were significantly increased compared with control rats. While in valsartan-treated group, the mRNA expressions were decreased compared to the DCM group (see Fig. 6).

## Conclusions

The DCM is associated with a significant myocardial remodeling which consists of cardiac hypertrophy, interstitial fibrosis, and changes in the cardiac metabolism [23]. Abundant evidences from experimental models of DCM indicated that the cardiac interstitial fibrosis and cardiac hypertrophy were the pathological substrate features of DCM [24, 25]. In the present study, Wister rats were made severely and chronically diabetic for 16 weeks with a single intraperitoneal injection of STZ (60 mg/kg body weight), and at the end of three weeks after STZ injection, the diabetic group rats were at a state of hyperglycemic, unfortunately, eight rats in diabetic group were died due to diabetic ketoacidosis, infection, fighting each other or digestive complications. In addition, there was one rat, which blood glucose does not meet the model standard, removed. Therefore, preventing acidosis caused by high blood sugar, keeping the environment clean and treating infections as well as other complications to ensure the quality and quantity of rats is very significant in this kind of trials. Moreover, changes in the cellular morphology and structural abnormalities of myocardium were found in the experiment. From HE and Masson’s staining of myocardium, it was found that the cardiomyocytes were disorganized with abundant myocardial collagen deposition, accompanied by a markedly increased left ventricular mass index. These salient endings of the present study demonstrated that 16 weeks of hyperglycemia contributes to a successful experimental Type 1 DCM with the development of cardiac hypertrophy and fibrosis.

Previous studies had demonstrated the pathophysiological role of periostin in myocardial fibrosis and cardiac remodeling *in vivo* and *in vitro*. Oka T, et al [26] found mice lacking the gene encoding periostin showed less fibrosis and better ventricular performance after a myocardial infarction, while inducible overexpression of periostin in the heart induced spontaneous hypertrophy with aging. In both animal and human models, periostin is closely associated with pressure overload-induced left ventricular hypertrophy (LVH) and LVH regression [9]. In vitro study found periostin is a profibrogenic matricellular protein that promotes collagen fibrogenesis, inhibits differentiation of progenitor cells into cardiomyocytes [27]. Periostin can also induce fibroblast proliferation and myofibroblast persistence in vitro [28]. In the present study, a high level of mRNA and protein expressions of periostin was found in the hearts of diabetic rats, consistent with the study of Zou P [15]. Periostin expression is localized to fibroblast, endothelial cells, inflammatory cells, and myocytes of the damaged heart. These results suggest that periostin might play an important role in the pathogenesis of DCM. Furthermore, in the DCM process, its myocardial cells metabolic disorders include glucose metabolism and lipid metabolism. The carbohydrate oxidation for energy is reduced and the use of lipid oxidation is increased as well as the material for the energy transferred from glucose to fatty acids, so the free fatty acid is elevated caused by lipid metabolism disorders has been widely accepted [29]. In addition, Carley et al [30] have shown that fatty acid, the endogenous ligand of Peroxisome Proliferator Activated Receptors α (PPARα), which uptake and utilization in the regulation of PPARα. Morever, the energy metabolism signaling pathway of PPARα-FFA in cardiomyocytes may joint with ventricular remodeling, which are important reasons for the pathogenesis of diabetic cardiomyopathy [31]. More study is needed to demonstrate if there is any relationship between FFA-metabolism and periostin on diabetic cardiomyopathy.

Experiment has demonstrated the existence and activation of a cardiac rennin-angiostensin system (RAS) in the hearts in diabetes [17]. In diabetic hearts, both the density and mRNA levels of Ang II receptors are increased [32, 33]. Blockage of RAS by Ang II receptor (AT1R) blockers prevents diabetes-induced heart dysfunction [34]. In our study, varstan, an AT1R antagonist, not only alleviated cardiac remodeling in DCM, but also reduced periostin expression. Together with the knowledge that Ang II could promote the mRNA and protein expression of periostin in vivo and in vitro [11], and periostin has been proved to take part in cardiac remodeling, we can conclude that valsartan could attenuate the cardiac remodeling in the DCM rats partly via down-regulating the expression of periostin. Periostin may become a new therapeutic target for DCM.

In addition, the present study data also revealed that the blood glucose levels of valsartan-treated diabetic rats were improved compared with diabetic rats. This result was accordant with the study of Chan P et al [35], which indicated that valsartan, not only decreased the systolic blood pressure but also improved the glucose utilization.

In conclusion, the present study findings confirmed up-regulated expression of periostin in DCM and provided strong evidence that valsartan protects against the cardiac remodeling in DCM rats which may partly related to the down-regulation of periostin protein, and provides a new insight into the potential therapeutic strategy of cardiac remodeling in DCM.

## Abbreviations

STZ: streptozototin
DCM: Diabetic cardiomyopathy
TGF-β1: Transforming growth factor
TGF-β1 R II: TGF-β1 type II receptor
CAD: Coronary artery disease
ECM: extracellular matrix
Ang II: Angiotensin II
RAS: Renin-angiostensin system
HE: Hematoxylin eosin
RT-PCR: Real-time polymerase chain reaction
LVH: Left ventricular hypertrophy
